# Fractional Diffusion, Low Exponent Lévy Stable Laws, and ‘Slow Motion’ Denoising of Helium Ion Microscope Nanoscale Imagery

**DOI:** 10.6028/jres.117.006

**Published:** 2012-02-22

**Authors:** Alfred S. Carasso, András E. Vladár

**Affiliations:** National Institute of Standards and Technology, Gaithersburg, MD 20899

**Keywords:** HIM images, ‘slow motion’ image denoising, image texture, image metrology, total variation, curvelet transform, fractional diffusion, low exponent Lévy stable laws, image Lipschitz exponents, surface morphology

## Abstract

Helium ion microscopes (HIM) are capable of acquiring images with better than 1 nm resolution, and HIM images are particularly rich in morphological surface details. However, such images are generally quite noisy. A major challenge is to denoise these images while preserving delicate surface information. This paper presents a powerful *slow motion* denoising technique, based on solving linear fractional diffusion equations forward in time. The method is easily implemented computationally, using fast Fourier transform (FFT) algorithms. When applied to actual HIM images, the method is found to reproduce the essential surface morphology of the sample with high fidelity. In contrast, such highly sophisticated methodologies as Curvelet Transform denoising, and Total Variation denoising using split Bregman iterations, are found to eliminate vital fine scale information, along with the noise. Image Lipschitz exponents are a useful image metrology tool for quantifying the fine structure content in an image. In this paper, this tool is applied to rank order the above three distinct denoising approaches, in terms of their texture preserving properties. In several denoising experiments on actual HIM images, it was found that fractional diffusion smoothing performed noticeably better than split Bregman *TV*, which in turn, performed slightly better than Curvelet denoising.

## 1. Introduction

This paper presents an easily implemented denoising methodology, based on solving linear fractional diffusion equations using fast Fourier transform (FFT) algorithms. The method conserves image *L*^1^ norms. When applied to state of the art nanoscale imagery, this method can outperform computationally more sophisticated denoising techniques based on *Curvelet Transform* thresholding [24], or on minimizing image *Total Variation* using the split Bregman iteration [13].

Both scanning He ion microscopes (HIM), and scanning electron microscopes (SEM), are capable of acquiring images with better than 1 nm resolution. Such imagery is very much needed in nano-scale research, development, and production. HIM images provide better surface-related information than SEM images, but are generally noisier. SEM images at the highest magnifications are also prone to noise. The important nano-structures are usually very small, and only the sharpest focused beams can resolve them. Such beams, formed with pA currents, involve a few hundred to a few thousand atoms and do not generate a lot of signal. This necessarily produces very noisy images. Any technique that can improve this situation is very much needed and would result in otherwise unavailable acquisition speed and/or information. However, preserving the fidelity of the essential sample-related fine details is of paramount interest.

This paper develops a useful *short time evolution* approach to this difficult problem. Fractional diffusion equations and related isotropic Lévy stable laws were introduced in image analysis in [4], where they were applied to solve an important class of image deblurring problems. Subsequently, it was discovered that *low exponent* Lévy stable laws could be successfully applied in blind deconvolution of a large variety of real blurred imagery of considerable scientific interest, including SEM images and Hubble space telescope as well as other astronomical images [5], [7–8]. In such deblurring applications, one solves *ill-posed* fractional diffusion equations backward in time, with the blurred image as data at time *t* = 1. In contrast, the present denoising application involves solving well-posed linear equations forward in time up to some small *t*^†^ > 0, with the noisy image as data at time *t* = 0. In that context, the significance of low Lévy exponents is expressed by the sharp inequality developed in Eq. (13). That inequality suggests that for small *t* > 0, low Lévy exponent fractional diffusion smoothing retains considerably more of the fine structure in the initial data, than does Gaussian smoothing, which corresponds to smoothing with the classical heat conduction equation. In Secs. 12 and 13, this expectation is borne out in a documented comparison of short time smoothing of HIM images, using various diffusive evolution equations.

In an entirely different direction, there has been considerable interest in recent years in the use of nonlinear anisotropic diffusion equations in image denoising [1], [16], [19–21]. The *Total Variation* (*TV*) approach is the best-known example of this class of methods. Here, the denoised image is defined to be the *long time steady-state* solution in a nonlinear diffusion equation with an inhomogeneous forcing term [19], [21]. Up to date surveys of *TV* denoising methodologies together with useful software, may be found in [11] and [13]. The split Bregman iteration is a particularly effective method of reaching the desired steady state. The *TV* approach is especially useful when the ideal sharp image is piecewise smooth, and consists of isolated smooth objects with well-defined edges. Such images belong to the class *BV*(*R*^2^) of functions of *bounded variation*, which plays an essential role in this theory. Important examples of effective *TV* denoising of such images, in the field of medical computed tomography, are discussed in [27].

Another important class of methods centers on wavelet transforms, and filtering the image by appropriately thresholding the wavelet coefficients. Recently, a more effective approach has been developed, based on the use of *Curvelets* that can better represent curved edges in the image [3], [24]. In [22], curvelet transform denoising is again successfully applied to computed tomography brain slices.

While both *TV* and Curvelet denoising work quite well on *BV*(*R*^2^) images, many important classes of images *f*(*x*, *y*) display significant fine scale details or *texture*, together with amorphous features, and do not belong to *BV*(*R*^2^) [6], [14]. Use of *TV* or Curvelet processing of such imagery may eliminate texture along with the noise, while preserving edges. The *L*^1^ Lipschitz exponent *α*, where 0 < *α* ≤ 1, is a mathematical index that can capture the fine-structure content and degree of unsmoothness in an image, provided that image is relatively *noise free*. Images that are of bounded variation (including smoothly differentiable images) have *α* = 1. The value of *α* decreases systematically with increasing roughness. Images with significant non differentiable small scale structures typically have *α* ≪ 1. A method of estimating image Lipschitz exponents is developed in [6] and applied to image restoration in [9].

HIM images are examples of images where texture and detailed surface morphology are of prime interest, and for which *TV* and Curvelet denoising may not be appropriate. In this paper, we exhibit several examples of real HIM data, where fractional diffusion denoising is superior to both Curvelet and split Bregman *TV* denoising, in retaining essential sample-related features. These improvements may not be visually apparent in the reduced size images in a printed copy of this paper. However, significant enhancement becomes evident when the *on-line* version of this paper is viewed at full size on a modern high resolution device, such as a wide screen, active matrix, liquid crystal display (LCD) monitor. By estimating image Lipschitz exponents *α* before and after denoising, we can use *α* as a useful metric to rank order these three distinct denoising methods in their ability to retain texture, and confirm the visual results.

## 2. Fourier Space Characterization of Textured Imagery

For purposes of theoretical analysis it is helpful to think of images *f*(*x*, *y*) as functions in *L^p^*(*R*^2^), *p* = 1, 2, i.e., functions such that

(1) $${\left\|f\right\|}_{p}={\left\{\int_{R^{2}}{\left|f(x,y)\right|}^{p}dxdy=\right\}}^{1/p}<\infty ,\;p=1,2.$$

Define the 2D Fourier transform of *f*(*x*, *y*) by

(2) $$F\{f\}=\hat{f}(\xi ,\eta )\equiv \int_{R^{2}}f(x,y)\exp\{-2\pi i(\xi x+\eta y)\}dxdy.$$

The *L*^1^ norm of *f*(*x*, *y*) is proportional to the total image radiant flux, and conservation of ║*f*║_1_ is a desirable attribute in any image processing method. Since *f*(*x*, *y*) ≥ 0, it follows from Eq. (2) that $\hat{f}(0,0)={\left\|f\right\|}_{1}$.

Let $\left|\nabla f\right|={\left(f_{x}^{2}+f_{y}^{2}\right)}^{1/2}$. Assume |∇*f*|∈*L*^1^(*R*^2^)∩*L*^2^(*R*^2^) so that both ║∇*f*║_1_ and ║∇*f*║_2_ are finite. Then, from Parseval’s theorem

(3) $$\int_{R^{2}}{\left|\nabla f\right|}^{2}dxdy=\int_{R^{2}}\left(f_{x}^{2}+f_{y}^{2}\right)dxdy=4\pi^{2}\int_{R^{2}}\left(\xi^{2}+\eta^{2}\right){\left|\hat{f}(\xi ,\eta )\right|}^{2}d\xi d\eta <\infty .$$

This implies that $\left|\hat{f}(\xi ,\eta )\right|$ must decay sufficiently fast at infinity to make the last integral converge. Images *f*(*x*, *y*) with significant fine structure need not satisfy the assumption |∇*f*|∈*L*^1^(*R*^2^)∩*L*^2^(*R*^2^), and both ║∇*f*║_1_ and ║∇*f*║_2_, may be infinite. In that case, $\left|\hat{f}(\xi ,\eta )\right|$ does not decay fast enough at infinity.

## 3. Linear Fractional Diffusion Equations and Lévy Stable Denoising

For fixed *β* with 0 < *β* ≤ 1, consider the linear fractional diffusion initial value problem in *L*^2^(*R*^2^),

(4) $$w_{t}=-{\left(-\Delta \right)}^{\beta }w,\;t>0,\;w(x,y,0)=f(x,y),$$

where Δ denotes the 2D Laplacian. This reduces to the classical heat equation when *β* = 1. Eq. (4) has the unique Fourier space solution

(5) $$\hat{w}(\xi ,\eta ,t)=\exp\left\{-t{\left[{\left(2\pi \xi \right)}^{2}+{\left(2\pi \eta \right)}^{2}\right]}^{\beta }\right\}\hat{f}(\xi ,\eta ),t>0,$$

from which *w*(*x*, *y*, *t*) can be found by inverse Fourier transformation

(6) $$w(x,y,t)=\int_{R^{2}}\exp\{2\pi i(\xi x+\eta y)\}\exp\left\{-t{\left[{\left(2\pi \xi \right)}^{2}+{\left(2\pi \eta \right)}^{2}\right]}^{\beta }\right\}\hat{f}(\xi ,\eta )d\xi d\eta .$$

In Eq. (5), the function

(7) $$\hat{h}(\xi ,\eta ,t)=\exp\left\{-t{\left[{\left(2\pi \xi \right)}^{2}+{\left(2\pi \eta \right)}^{2}\right]}^{\beta }\right\},\;t>0,$$

is the Fourier transform of the Green’s function for the linear fractional diffusion equation in Eq. (4). For each fixed *t* > 0, the function in Eq. (7) is also the Fourier transform of an *isotropic Lévy stable probability density function* with exponent 2*β*, [12], [18], [23]. In physical (*x*, *y*) space, such probability densities are bell-shaped functions with increasingly heavy tails as *β* decreases from *β* = 1. The choice *β* = 1 corresponds to the Gaussian density, while *β* = 1/2 corresponds to the Lorentzian density. For other values of *β*, the corresponding density is not known in closed form in physical (*x*, *y*) space. As *t* decreases, these functions become steeper and narrower, approaching the 2D Dirac δ-function as *t* ↓ 0. In the image deblurring applications discussed in [5], [7], [8], such Lévy stable laws play a vital role as candidate *point spread functions*. In the present application, denoising is accomplished by effectively blurring the noisy image with such narrow point spread functions.

We note that from Eq. (5), $\hat{w}(0,0,t)=\hat{f}(0,0)$, *t* > 0. Hence,

(8) $${\left\|w(.,t)\right\|}_{1}={\left\|f\right\|}_{1},\;t>0,$$

and the linear diffusion smoothing process in Eq. (4) conserves the image *L*^1^ norm.

## 4. Monotonicity And The Significance Of Low *β* Values

In the Hilbert space *L*^2^(*R*^2^), the unique solution *w*(*x*, *y*, *t*) in Eq. (6) satisfies

(9) $${\left\|w(.,t)-f\right\|}_{2}^{2}=\int_{R^{2}}{\left\{1-\exp\{-t{\left[{\left(2\pi \xi \right)}^{2}+{\left(2\pi \eta \right)}^{2}\right]}^{\beta }\}\right\}}^{2}{\left|\hat{f}(\xi ,\eta )\right|}^{2}d\xi d\eta .$$

Hence,

(10) $${\left\|w(.,t_{2})-f\right\|}_{2}\geq {\left\|w(.,t_{1})-f\right\|}_{2},\;t_{2}\geq t_{1}\geq 0.$$

At the same time, from Eq. (3), |∇*w*(*x, y, t*)| satisfies

(11) $${\left\|\nabla w(.,t)\right\|}_{2}^{2}=4\pi^{2}\int_{R^{2}}(\xi^{2}+\eta^{2})\exp\left\{-2t{\left[{\left(2\pi \xi \right)}^{2}+{\left(2\pi \eta \right)}^{2}\right]}^{\beta }\right\}{\left|\hat{f}(\xi ,\eta )\right|}^{2}d\xi d\eta .$$

Hence,

(12) $${\left\|\nabla w(.,t_{2})\right\|}_{2}\leq {\left\|\nabla w(.,t_{1})\right\|}_{2},\;t_{2}\geq t_{1}\geq 0.$$

If |∇*f*|∈*L*^1^(*R*^2^), then ║∇*w*(.,*t*)║_2_ ≤ ║∇*f*║_2_, *t* ≥ 0. If |∇*f*|∉*L*^2^(*R*^2^), then ║∇*w*(.,*t*)║_2_ is finite for *t* > 0, but becomes infinite as *t* ↓ 0. The rate at which this happens depends on *β*, as ║∇*w*(.,*t*)║_2_ = *O*(*t*^−1/2^*^β^*), *t* ↓ 0. In fact, with *e* = 2.71828…, the following sharp inequality is a consequence of Eq. (11),

(13) $${\left\|\nabla w(.,t)\right\|}_{2}\leq \underset{\rho \geq 0}{\sup}\{\rho \exp(-t\rho^{2\beta })\}{\left\|f\right\|}_{2}{=\{2\beta te\}}^{-1/2\beta }{\left\|f\right\|}_{2},\;t>0.$$

In the case of Gaussian smoothing, corresponding to *β* = 1, this inequality implies ║∇*w*(.,*t*)║_2_ = *O*(*t*^−1/2^), as *t* ↓ 0. In contrast, ║∇*w*(.,*t*)║_2_ = *O*(*t*^−5^), as *t* ↓ 0 when *β* = 0.1. This suggests that for small *t* > 0, the solution. *w_β_*(*x*, *y*, *t*), with *β* ≪ 1, retains considerably more of the small scale features in the initial data *f*(*x*, *y*), than is the case with Gaussian smoothing. This point is explored further in Secs. 12 and 13.

## 5. Computational Considerations

We deal exclusively with square images *g*(*x*, *y*) of size 2*N* × 2*N* pixels, and the fast Fourier transform (FFT) is the primary computational tool used in this paper. In order to render mathematical formulae more transparent, we use the same notation, *ĝ*(*ξ*, *η*), for both discrete and continuous Fourier transforms. In the discrete FFT case, the frequencies 2*πξ* and 2*πη* are understood to be integer-valued and to range from −*N* to *N*. Likewise, *g*(*x*, *y*) denotes both discrete and continuous images. In the discrete case, the variables *x*, *y* are measured in pixels and range from 1 to 2*N*.

We may compute the solution *w*(*x*, *y*, *t*) in Eq. (4) at any given *t* > 0, by using the forward and inverse FFT to implement the operations in Eq. (5) and Eq. (6) respectively. However, a more efficient recursive procedure can be used. With *ĥ*_(_*ξ*, *η*, *t*) as in Eq. (7), let *ϖ* = 1.0/*K* for a fixed positive integer *K* sufficiently large, and define *Q*(*ξ*, *η*) = *ĥ*(*ξ*, *η*, *ϖ*). With $\hat{w}(\xi ,\eta ,0)=\hat{f}(\xi ,\eta )$, consider the following recursion

(14) $$\hat{w}(\xi ,\eta ,k\varpi )=Q(\xi ,\eta )\hat{w}(\xi ,\eta ,(k-1)\varpi ),\;k=1,2,3,\cdots .$$

An inverse FFT in Eq. (14) at each integer *k*, generates the solution *w*(*x*, *y*, *t*) at as many discrete times *t_k_* = *kϖ*, as desired. Diagnostic statistical information about *w*(*x*, *y*, *t*) can also be calculated for selected values of *t_k_* as *t* increases. Of particular interest are the discrete *L^p^* norms, *p* = 1, 2, defined as follows

(15) $${\left\|w_{d}(.,t)\right\|}_{p}={\left\{{\left(2N\right)}^{-2}\sum_{x,y=1}^{2N}{\left|w(x,y,t)\right|}^{p}\right\}}^{1/p}.$$

As in Eq. (8), we have ║*w_d_*(.,*t*)║_1_ = ║*w_d_*(.,0)║_1_, *t* > 0. We also define the discrete analogs of ║∇*w*(.,*t*)║*_p_*, *p* = 1, 2, as follows

(16) $${\left\|\nabla_{d}w(.,t)\right\|}_{p}={\left\{{\left(2N\right)}^{-2}{\sum_{x,y=1}^{2N-1}\left({\left\{w_{x}\left(x,y,t\right)\right\}}^{2}+{\left\{w_{y}\left(x,y,t\right)\right\}}^{2}\right)}^{p/2}\right\}}^{1/p}.$$

where

(17) $$\begin{array}{l} w_{x}(x,y,t)={\left(2N\right)}^{-1}(w(x+1,y,t)-w(x,y,t)),\\ w_{y}(x,y,t)={\left(2N\right)}^{-1}(u(x,y+1,t)-u(x,y,t)). \end{array}$$

Note that for a mathematical image *f*(*x*, *y*) ∈ *L^p^* (*R*^2^) as considered in Sec. 2, we may have ║∇*f*║*_p_* = ∞. However, the corresponding 2*N* × 2*N* pixels image *f*(*x*, *y*) will have a finite value for ║∇*_d_ f*║*_p_*, although that value will be relatively large.

A minimum principle for short time fractional diffusion smoothing will be given in Sec. 8. It is helpful to set the stage for this by first discussing *TV* and Curvelet denoising.

## 6. Split Bregman Iterative Total Variation (*TV*) Denoising

A function *u*(*x*, *y*) is in *BV*(*R*^2^) if

(18) $$\int_{R^{2}}\left|Du\right|+{\left\|u\right\|}_{1}<\infty ,$$

Where *Du* is the distribution gradient of *u*(*x*, *y*) [1]. However, in actual numerical computation [10], [13], [17], the *isotropic* discrete *TV* seminorm defined by

(19) $${\left\|u\right\|}_{TV}={\left\|\nabla_{d}u\right\|}_{1},$$

with ║∇*_d_u*║_1_ as in Eq. (16) above, is widely used. Given a noisy image *f*(*x*, *y*), and the regularization parameter *ω* > 0, total variation denoising seeks a function *f ^T^*(*x*, *y*) such that

(20) $$f^{T}(x,y)=Arg\underset{u\in BV(R^{2})}{\min}\left\{{\left\|\nabla_{d}u\right\|}_{1}+\omega /2{\left\|u-f\right\|}_{2}^{2}\right\}.$$

There are several methods that can be used to solve this minimization problem [10–11], [13], [19]. The method in [19] obtains *f ^T^*(*x*, *y*) as the unique steady-state solution to a nonlinear anisotropic diffusion equation with *f*(*x*, *y*) as initial data. A more effective method is the split Bregman iteration discussed in [13]. In this paper, we apply a MATLAB implementation of the isotropic split Bregman approach, as developed by the authors in [13], to denoise HIM images. Split Bregman *TV* denoising of *f*(*x*, *y*) typically reduces ║∇*_d_ f*║_1_ considerably and need not preserve ║*f*║_1_.

## 7. Curvelet Denoising

The curvelet transform is designed to represent edges and other singularities along curves, more efficiently than traditional wavelet methods [3], [24]. In this paper we use a MATLAB implementation of curvelet denoising developed by the authors in [24], and made available in their CurveLab package. The basic idea is discussed in [24, Sec. 5]. The noisy image *f*(*x*, *y*) is assumed corrupted by white noise with a noise level *σ_n_*. Let *W_λ_* be the noisy curvelet coefficients corresponding to *f*(*x*, *y*). The code estimates the variances *σ_λ_* of the coefficients *W_λ_*, using knowledge of *σ_n_* together with a Monte Carlo simulation that estimates the *L*^2^ norms of individual curvelets. The denoised image curvelet coefficients ${\bar{W}}_{\lambda }$ are then obtained by thresholding the noisy coefficients as follows

(21) $${\bar{W}}_{\lambda }=W_{\lambda },\;\text{if}\;\left|W_{\lambda }\right|/\sigma_{n}\geq k\sigma_{\lambda },\;{\bar{W}}_{\lambda }=0,\;\text{if}\;\left|W_{\lambda }\right|/\sigma_{n}\geq k\sigma_{\lambda }.$$

Here, *k* = 3, except for the finest scale, where *k* = 4. As in the *TV* case, Curvelet denoising significantly reduces ║∇*_d_ f*║_1_ and need not preserve ║*f*║_1_.

The Curvelet denoising experiments discussed in [3], [24], and [22], involve synthetically degraded images, where both the noise level *σ_n_*, as well as the type of noise, are known. In our experiments below on real HIM images, such knowledge is unavailable, and we must use educated guesses for the input *σ_n_*.

## 8. Minimum Principle for ‘Slow Motion’ Lévy Stable Denoising

Given a noisy image *f*(*x*, *y*), calculate ║∇*_d_ f*║*_p_*, *p* = 1, 2. Fix *β*, with 0 < *β* < 1/2, and consider the evolution problem

(22) $$w_{t}=-{\left(-\Delta \right)}^{\beta }w,t>0,\;w(x,y,0)=f(x,y).$$

As previously indicated, using the recursion in Eq.(14) and FFT algorithms, we may readily solve Eq. (22) and calculate ║∇*_d_ w*(.,*t*)║*_p_*, = *p* = 1, 2, for selected *t* values as *t* increases. Analogously to the minimum principle in Eq. (20), we pose the following minimization problem. For given fixed *λ* with 0 < *λ* < 1,

(23) $$f^{L}(x,y)=\mathrm{Arg}\;\underset{t>0}{\min}\left\{{\left\|w(.,t)-f\right\|}_{2}∍{\left\|\nabla_{d}w(.,t)\right\|}_{2}\leq \lambda {\left\|\nabla_{d}f\right\|}_{2}\right\}.$$

**Fig. 1:**
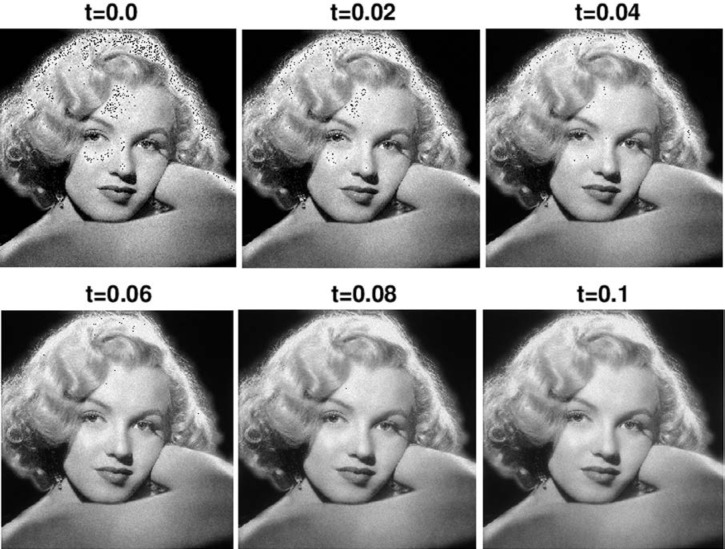
Poisson noise degraded Marilyn Monroe image *f*(*x*, *y*) at *t* = 0, is progressively denoised using the recursion in Eq. (14) to solve Eq. (22) from *t* = 0 to t^†^ = 0.1, with *β* = 0.2. In that time interval, for *p* = 1, 2, ║∇*_d_w*(., *t*)║*_p_*, decreases monotonically by a factor *λ* ≈ 0.46, while ║*w_d_*(.,*t*)║_1_ is conserved.

In view of the monotonicity results in Eq. (10) and Eq. (11), this minimum principle has the unique solution *f ^L^*(*x*, *y*) = *w*(*x*, *y*, *t*^†^), where *t*^†^ > 0 is the earliest time at which ║∇*_d_ w*(.,*t*)║_2_≤ *λ* ║∇*_d_ f*║_2_. As shown in Fig. 1, one can monitor this denoising process as *t* increases from *t* = 0 to t = *t*^†^, by displaying the image evolution, and evaluating the accompanying diagnostic information, ║*w^d^* (.,*t*)║*_p_* and ║∇*_d_ w*(.,*t*)║*_p_*, *p* = 1, 2. In Fig. 1, a 512 × 512 pixel Marilyn Monroe image, degraded by signal-dependent Poisson noise, is used as initial datum in Eq. (22) with *β* = 0.2. Initially, ║∇*_d_ f*║_1_ = 11,000, ║∇*_d_ f*║_2_= 15,000. These gradients decay monotonically to the values 5000 and 6900, respectively, at *t* = 0.1, while ║*w_d_* (.,*t*)║_1_ is conserved. Thus, *t* = 0.1 would correspond to *t*^†^, had *λ* been chosen to be ≈ 0.46. Such displays enable the user to decide whether important small-scale information has been smoothed out, along with the noise, prior to reaching *t*^†^. The process can then be restarted with different values of *β* and *λ*. These are valuable exploratory options in practice. Such options are unavailable in the Total Variation method which, given the regularization parameter *ω*, produces a single final denoised image, defined as the limit of the convergent Split Bregman iteration in [13], or as the steady-state solution in the nonlinear anisotropic diffusion problem in [19]. Likewise, given the input noise level *σ_n_*, curvelet denoising results in a single final denoised image.

## 9. Image Fine Structure and Lipschitz Exponents

Most natural images *f*(*x*, *y*) are not smoothly differentiable functions of *x* and *y*, but display edges, localized sharp features, and other significant fine scale details or *texture*. The image Lipschitz exponent measures the fine structure content of an image, provided that image is relatively noise free [9]. An image *f*(*x*, *y*) has *L*^1^ Lipschitz exponent *α*, if and only if

(24) $$\int_{R^{2}}\left|f(x+h_{1},y+h_{2})-f(x,y)\right|dxdy\leq \mathrm{Const}{\left|h\right|}^{\alpha },\;\left|h\right|\to 0,$$

where $\left|h\right|={\left(h_{1}^{2}+h_{2}^{2}\right)}^{1/2}$, and *α* is fixed with 0 < *α* ≤ 1. An image that is of bounded variation, or smoother, has *α* = 1. The value of *α* decreases with increasing fine structure. Most natural images have *α* < 0.6, and are not of bounded variation.

In [6], [9], an effective method for estimating image Lipschitz exponents is developed, based on a major theorem in [26]. See also [2], [25]. For fixed *τ* > 0, define the linear operator *G^τ^* by means of the Fourier series

(25) $$\{G^{\tau }f\}(x,y)=\sum_{m,n=-\infty }^{\infty }\exp\{-\tau (m^{2}+n^{2})\}{\hat{f}}_{mn}\exp\{2\pi i(xm+yn)\},$$

where ${\hat{f}}_{mn}$ are the Fourier coefficients of the image *f*(*x*, *y*), the latter assumed defined on the unit square. Let *μ*(*τ*) be the *L*^1^ relative error in approximating *f*(*x*, *y*) with the Fourier series {*G^τ^ f*}(*x*, *y*),

(26) $$\mu (\tau )={\left\|G^{\tau }f-f\right\|}_{1}/{\left\|f\right\|}_{1},\;\tau >0.$$

As shown in [26], an image *f*(*x*, *y*) has Lipschitz exponent *α* if and only if *μ*(*τ*) =O(*τ^α^*^/2^) as *τ* ↓ 0. Because of the exponential decay, the infinite series in Eq. (25) can be well-approximated by a finite sum for each fixed *τ*. Such a sum can be formed using FFT algoritms, and *G^τ^ f* can be evaluated for each fixed *τ_n_* > 0 in a sequence {*τ_n_*} tending to zero, together with *μ*(*τ_n_*). By plotting *μ*(*τ_n_*) vs *τ_n_* on a log-log scale, positive constants *C* and *α* can be located such that *μ*(*τ*) ≤ *C τ^α^*^/2^ as *τ* ↓ 0.

**Fig. 2:**
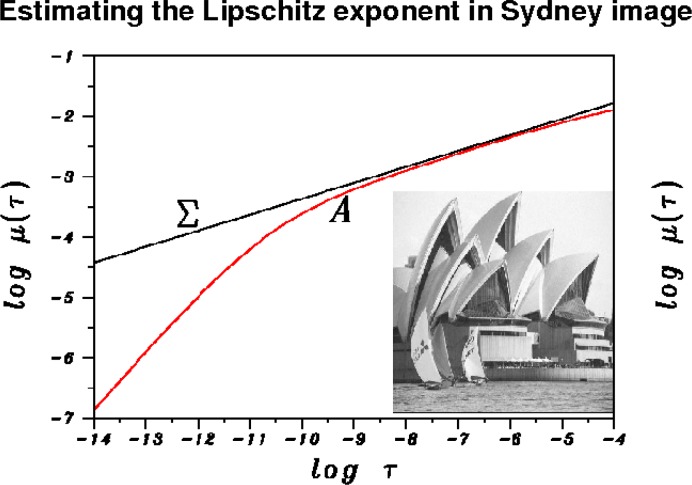
With *μ*(*τ*) as in Eq. (26), red curve *A* is a plot of *μ*(*τ*) versus *τ*, on a log-log scale. Image Lipschitz exponent equals twice the slope of majorizing line Σ. That slope is 0.265, indicating that the Sydney image has *L*^1^ Lipschitz exponent *α* = 0.530.

Figure 2 describes this Lipschitz estimation procedure as applied to a 512 × 512 pixels Sydney image *f*(*x*, *y*). The above FFT procedure was used to obtain *μ*(*τ_n_*) in Eq. (26) at 400 values *τ_n_* = 0.5(0.95*^n^*, *n* = 1,400. A plot of *μ*(*τ*) versus *τ* on a log-log scale produced the red curve *A* in Fig. 2. The curve *A* exhibits a characteristic *elbow shape*. It consists of a straight line segment with slope ≈ 1, beginning near log *τ* = −15 and continuing to near log *τ* = −10. There is then a transition to a different regime, one that is more slowly increasing and that continues to near log = 0. As explained more fully in [6], [9], the rapidly varying portion for log *τ* < −10 is a fallacious finite-dimensionality artifact, unrelated to the true image Lipschitz exponent. Only the slowly varying part of *A* is relevant to estimating Lipschitz exponents. Least squares fitting on −9 ≤ log *τ* ≤ −4 was used to find the majorizing straight line Σ for the slowly varying part of the red curve *A*. The *y*-axis intercept value obtained by least squares was slightly increased so as to make the line Σ lie visibly above the red curve *A*. However, the slope of Σ remains the same as that obtained from least squares. The line Σ is defined by log *μ*(*τ*) = −0.902 + 0.265 log *τ*, implying that *μ*(*τ*) ≤ 0.493 *τ*^0.265^ as *τ* ↓ 0. According to the theorem in [26], the Sydney image has Lipschitz exponent *α* = 0.530.

## 10. Noise Contamination, Smoothing, and Image Lipschitz Exponents

**Fig. 3:**
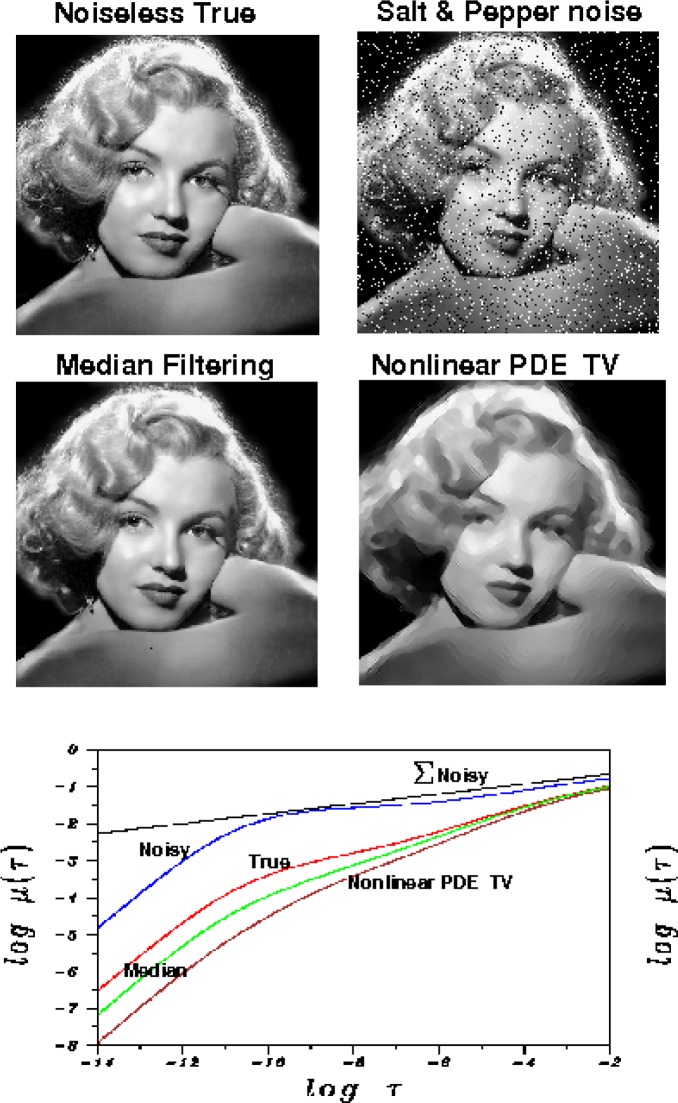
Adding noise to an image artificially decreases the Lipschitz exponent, but some forms of denoising can eliminate texture along with the noise and increase the Lipschitz exponent. Salt and pepper noisy image (blue curve) has a substantially smaller Lipschitz exponent *α* = 0.260, than the noiseless true image (red curve) with *α* = 0.594. *TV* denoising using long time steady-state solution in the nonlinear pde in Reference [19] (brown curve), leads to a Lipschitz exponent *α* = 0.812, substantially higher than the noiseless true image. Better results are obtained with median filtering (green curve) with a Lipschitz exponent *α* = 0.714.

The behavior of image Lipschitz exponents *α* before and after processing, is of major interest. As shown in Fig. 3, an original sharp, noiseless, 512 × 512 pixel Marilyn Monroe image *f*(*x*, *y*), has *α* = 0.594 and ║∇*_d_ f*║_1_= 5600. This is synthetically contaminated by addding salt and pepper noise with density 0.1. Such noise artificially decreases *α* to the value *α* = 0.260, while simultaneously increasing ║∇*_d_ f*║_1_ to the value 22000. As previously noted, the Lipschitz exponent is a true measure of image fine structure, only if that image is relatively noise free. A variety of algorithms may now be applied to denoise the degraded Marilyn Monroe image, with varying degrees of success. Some algorithms may smooth out genuine fine details along with the noise, decreasing ║∇*_d_ f*║_1_ and simultaneously increasing *α*, often well beyond their true values in the original noiseless image. This is the case with the *TV* denoised image in Fig. 3, which has ║∇*_d_ f*║_1_ = 2010, and *α* = 0.812. Here, a procedure different from the split Bregman method was used for *TV* denoising. This is the previously mentioned approach in [19], where the denoised image corresponds to the steady-state solution in a nonlinear anisotropic diffusion equation. The finite difference scheme in [19] was used with Δ*t* = 0.1(Δ*x*)^2^, regularization parameter Λ = 2.0, and forward integration carried for 300 time steps Δ*t*. Better results are obtained with median filtering using a 3 × 3 neighborhood, leading to a Lipschitz exponent of 0.714. In Fig. 3, least squares fitting on −10 ≤ log *τ* ≤ 0 was used to find the majorizing Σ lines. These observations will be helpful in the next section.

## 11. Denoising State of the Art HIM Imagery

The Helium Ion Microscope (HIM) is a new type of microscope that works by scanning a well-focused He ion beam over the surface of the sample in a raster pattern. The technique is very similar to the method used in scanning electron microscopes (SEM). As for SEM, the most significant signal is produced by the secondary electrons (SE), especially the SE1 electrons that are generated right at the point where the charged beam hits the sample. These electrons carry information about the finest morphological details of the sample. For HIM, more of these electrons are produced than in SEM, with a higher proportion of SE1 and other SE signals. Consequently, the resulting secondary electron images are richer in surface details. The price for this is a generally somewhat worse signal-to-noise ratio. The He beam current is typically smaller, and even the use of sub-pA current is feasible. With certain samples it is recommended to use low beam currents to avoid the erosion of the samples.

The resulting higher noise levels hamper the extraction and interpretation of needed morphological information from HIM images. While efforts to further improve resolution are currently focused on new hardware designs, it is important to explore and develop software-based solutions as well. One solution lies in denoising HIM and SEM images, which when done well, can substantially improve image quality. High-frequency components of the signal carry information about fine details, sharp edges, and fast transition in grey levels. Imaging instruments typically have a transfer function that shows worsening signal-to-noise ratio for fine details, because the noise is commonly worse in the high-frequency range. Simple denoising methods that merely delete the high Fourier frequency portion of the signal are not helpful. Methods that introduce unacceptably large distortions, or lead to significant blurring, are not very valuable either, especially for measurement purposes. Many of the currently available denoising methods fall into this category. However, the split Bregman total variation minimization method, and the Curvelet Thresholding method, are two highly sophisticated denoising methods that have undergone intensive development over the last ten years, and have found useful application in many areas [3], [13] [24]. Accordingly, it is appropriate to evaluate the fractional diffusion method on HIM imagery by comparing it with these two well-studied approaches.

The three 1024 × 1024 pixels HIM sample images discussed below, involve signal-dependent noise of unknown characteristics and intensity. Educated guesses must be used for the input regularization parameters. After some preliminary experimentation, a value of *ω* = 0.025 in Eq. (20) was selected for the split Bregman *TV* method, and a value *σ_n_* = 30 in Eq. (21) was adopted for the Curvelet Thresholding method. These values were used for all three samples, and they are well within the ranges recommended by the authors in [3], [13], [24], in the MATLAB implementations of their methods. In the fractional diffusion method, we used *β* = 0.2 in Eq. (4), and selected the solution at *t* = 0.1 in Eq. (14) as the denoised image in all three samples. This turned out to be equivalent to having chosen *λ* ≈ 0.33 in Eq. (23), so that *t*^†^ = 0.1.

**Fig. 4:**
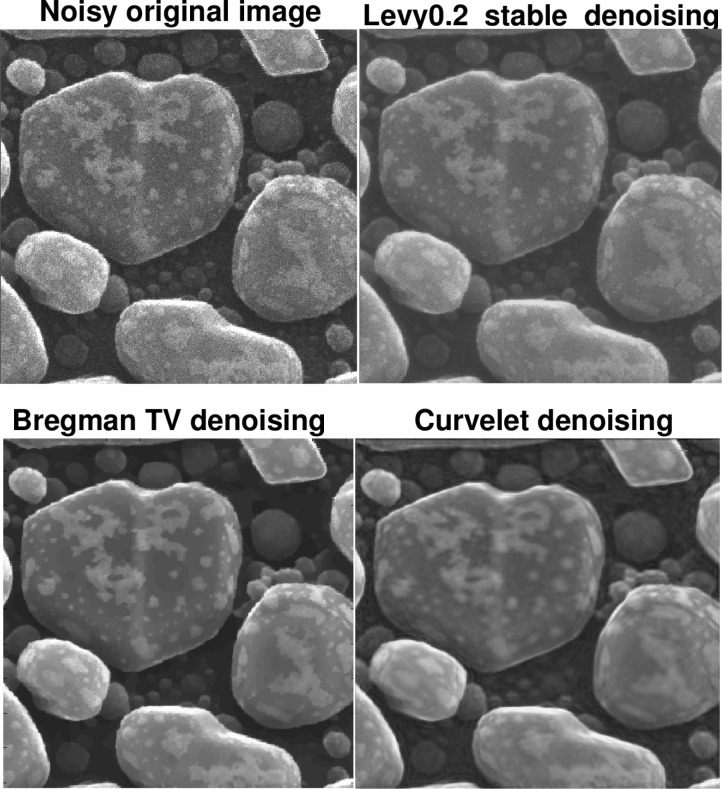
Denoising of actual HIM Au-decorated gold on carbon sample, using Lévy fractional diffusion, split Bregman TV, and Curvelet thresholding. Here, field of view is 600 nm. Important loss of structural detail is evident in TV and Curvelet images. This is confirmed by examining a portion of the sample as shown in Fig. 5, as well as the behavior of ║∇*_d_ f*║_1_ in Table 1, and the Lipschitz traces in Fig. 6. The original noisy image has Lipschitz exponent *α* = 0.241; the Lévy (*β* = 0.2) stable image has *α* = 0.451; the split Bregman TV image has *α* = 0.751; and the Curvelet image has *α* = 0.810.

**Fig. 5:**
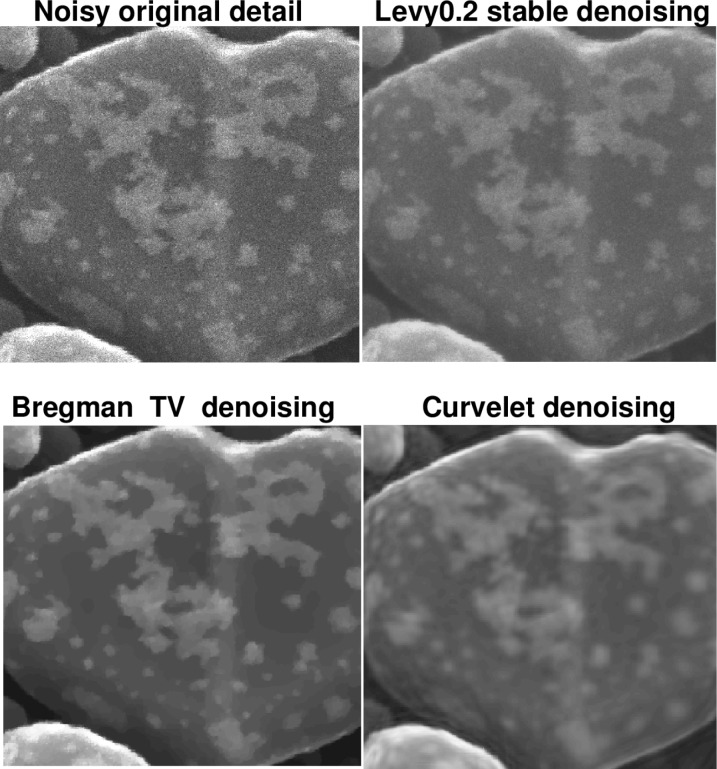
Poor recovery of surface morphology in TV and Curvelet denoising experiments on Au-decorated gold on carbon sample in Fig. 4. Here, field of view is 300 nm. This behavior is compatible with Table 1, and the Lipschitz traces in Fig. 6.

The following discussion presupposes access to the on-line version of this paper, together with a high-resolution computer screen. Figure 4 deals with an actual HIM image of an Au-decorated gold on carbon sample, with a field of view of 600 nm. In Fig. 4, the Lévy (*β* = 0.2) denoised image has maintained fidelity to the surface texture in the foreground, as is evident from the jagged edges, as well as to the small structures in the backgound. Such background structures are not well-recovered in the *TV* denoised image, while the foreground surface texture has been smoothed. The background structures are better recovered in the Curvelet denoised image; however, the jagged edges in the foreground surface texture have now been eliminated. This becomes more evident when examining a portion of the sample, as shown in Fig. 5. In Table 1, we see that Lévy stable denoising conserves ║*f_d_*║_1_, while reducing ║∇*_d_ f*║_1_ by a factor of three. However, *TV* and Curvelet denoising do not conserve ║*f_d_*║_1_, and reduce ║∇*_d_ f*║_1_, by a factor of seven or more.

**Fig. 6:**
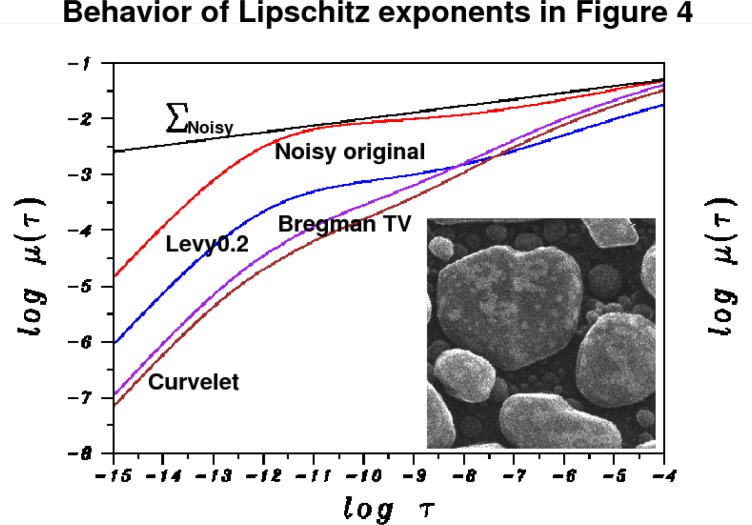
Behavior of *L*^1^ image Lipschitz exponents in denoising experiment on Au-decorated gold on carbon sample in Figs. 4 and 5. Here, only majorizing Σ line for original trace is shown. Remaining Σ lines are not drawn to avoid clutter. Red curve is original noisy trace with Lipschitz exponent *α* = 0.241. Blue curve is Lévy (*β* = 0.2) denoised trace with exponent *α* = 0.451. Purple curve corresponds to split Bregman TV trace, with substantially larger exponent *α* = 0.751. Brown curve is Curvelet trace with exponent *α* = 0.810. These values quantify the loss of fine-structure evident in Figs. 4 and 5.

The behavior of the *L*^1^ Lipschitz exponent *α* in Fig. 6 provides a useful metric in the denoising experiment in Figs. 4 and 5. The original noisy HIM image has *α* = 0.241, while the Lévy (*β* = 0.2) stable image has *α* = 0.451. The *TV* and Curvelet images have substantially higher values of *α*, namely 0.751 and 0.810, respectively. This is consistent with the behavior of ║∇*_d_ f*║_1_ in Table 1. In Fig. 6, as in all other 1024 × 1024 pixels HIM images discussed below, least squares fitting on −11 ≤ log *τ* ≤ −4 was used to find the majorizing Σ lines.

**Fig. 7:**
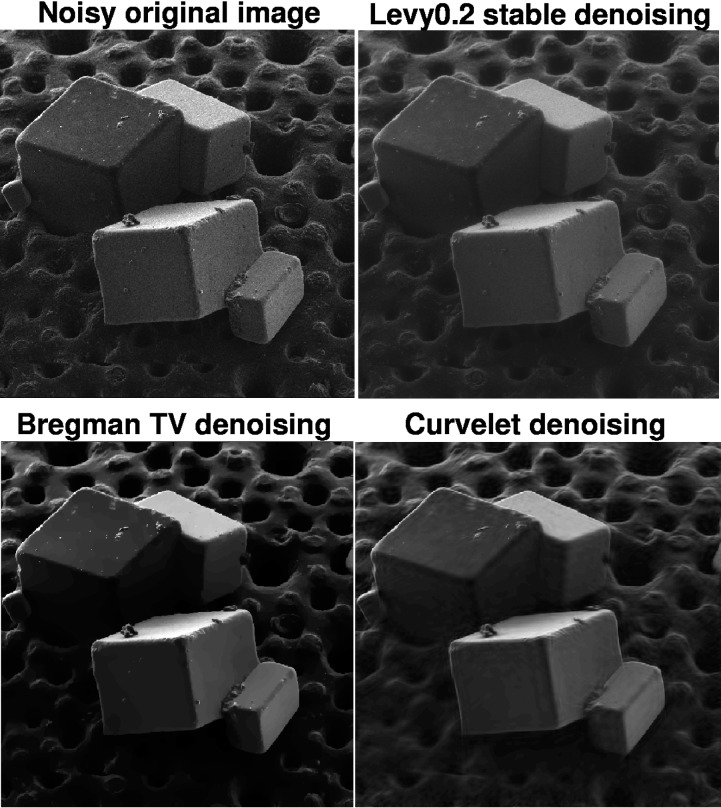
Denoising of actual HIM salt crystals on radiolaria sample, using Lévy fractional diffusion, split Bregman TV, and Curvelet thresholding. Here field of view is 90 *μ*m. Loss of structural detail is evident in TV and Curvelet images. This is confirmed by examining a portion of the sample as shown in Fig. 8, as well as the behavior of in Table 2. Note 30% drop in ║∇*_d_ f*║_1_, ║*f_d_*║_1_ after split Bregman TV processing, in Table 2. Lipschitz traces for this experiment (not shown), are very similar those in Fig. 6. Original noisy image has Lipschitz exponent *α* = 0.299. Lévy (*β* = 0.2) denoised image has *α* = 0.497. Split Bregman TV denoised image has *α* = 0.638. Curvelet denoised image has *α* = 0.668.

**Fig. 8:**
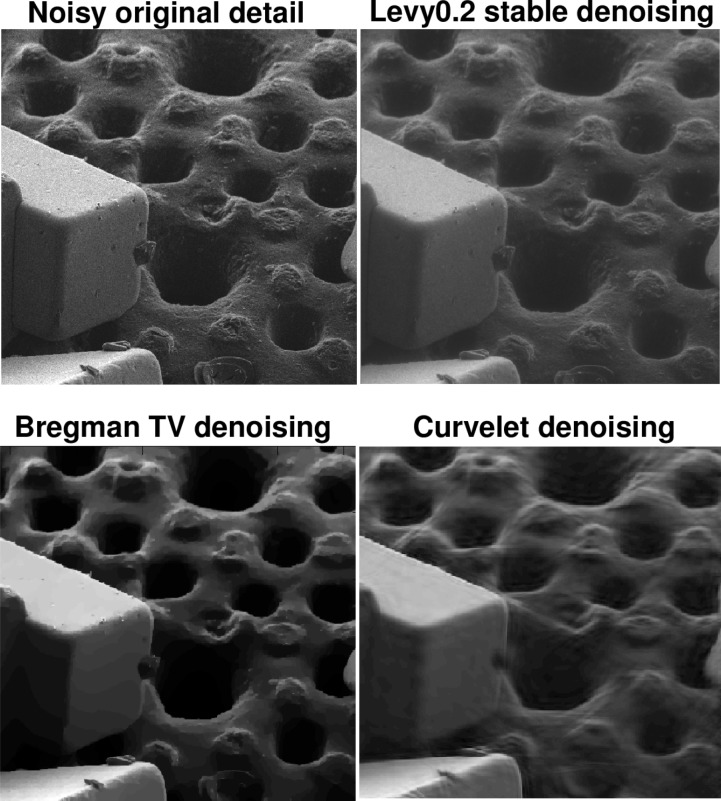
Examination of parts of the salt crystals on radiolaria sample in Fig. 7, under magnification, reveals significant erosion of surface morphology in TV and Curvelet images, as compared with Lévy stable image. Here, field of view is 45 *μ*m. Image Lipschitz exponents can quantify the loss of fine structure.

A very different sample image is considered in Fig. 7 but the denoising results show a similar pattern. Here, a salt crystal on radiolaria HIM image, with a field of 90 *μ*m is denoised. By examining a portion of the image in Fig. 8, it is evident that surface morphology is better reproduced in the Lévy (*β* = 0.2) stable image than it is in the *TV* and Curvelet images. The Lipschitz traces in this experiment (not shown) are very similar to those in Fig. 6. The original noisy HIM image has *α* = 0.299, while the Lévy (*β* = 0.2) stable image has *α* = 0.497. The *TV* and Curvelet images have higher values of *α*, namely 0.638 and 0.668, respectively. Again, Table 2 shows quite significant reductions in ║∇*_d_ f*║_1_ in the *TV* and Curvelet images, as compared with the Lévy stable image. In this example, the 30% drop in ║*f_d_*║_1_ after split Bregman *TV* processing, is striking.

**Fig. 9:**
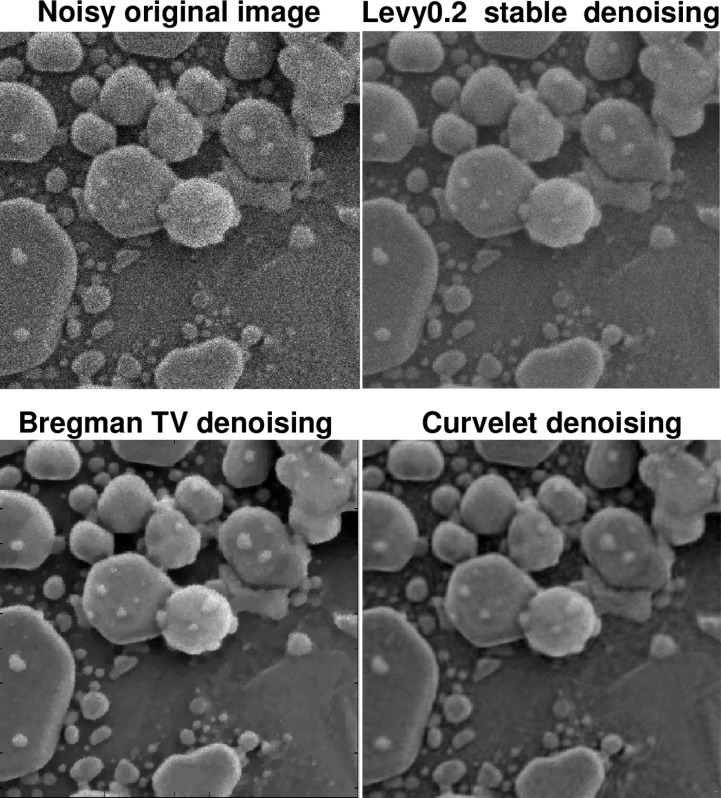
Denoising of actual HIM gold on carbon sample, using Lévy fractional diffusion, split Bregman TV, and Curvelet thresholding. Here, field of view is 300 nm. TV and Curvelet denoising produce smoother surfaces than Lévy stable denoising. However, true surfaces may not be smooth. Lipschitz traces for this experiment (not shown), are again very similar to those in Fig. 6. Here, the original noisy image has Lipschitz exponent *α* = 0.085; the Lévy (*β* = 0.2) stable image has *α* = 0.211; the split Bregman TV image has *α* = 0.697; and the Curvelet image has *α* = 0.704.

**Fig. 10:**
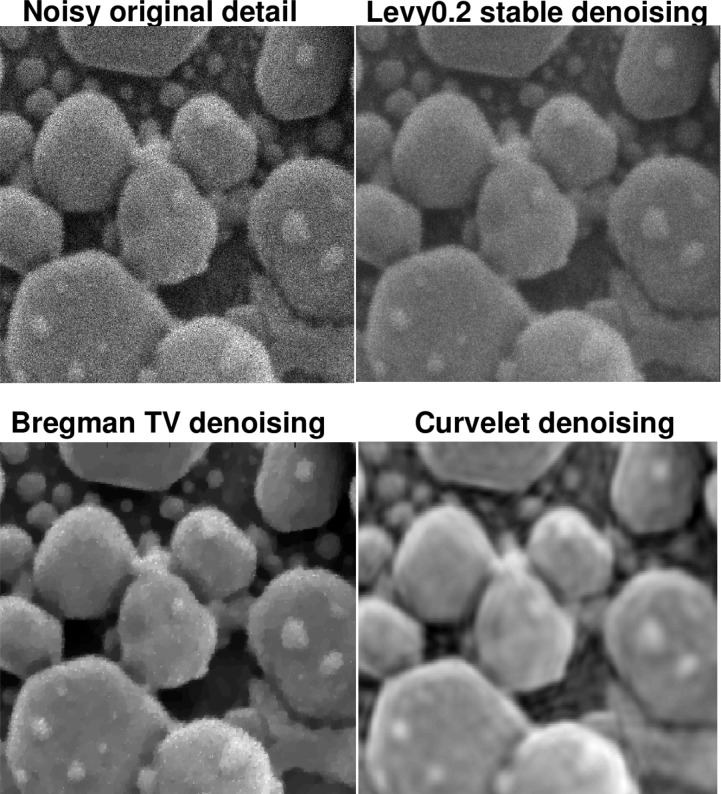
Examination of parts of the gold on carbon sample in Fig. 9, under magnification, reveals extent of TV and Curvelet smoothing of surface texture, as compared with Lévy stable image. Here, field of view is 150 nm.

The last example, in Figs. 9 and 10, is instructive. This is a gold on carbon sample, with a field of view of 300 nm. The original HIM image appears very noisy, and ║∇*_d_ f*║_1_ = 47000. Lévy (*β* = 0.2) stable denoising removes a considerable amount of noise, with ║∇*_d_ f*║_1_ reduced to 15000, but the resulting surfaces are still fuzzy. However, such surface fuzziness may be characteristic of the sample, much like the surface of a peach. The *TV* and Curvelet images exhibit aggressive denoising, resulting in ║∇*_d_ f*║_1_≤ 3500, and surfaces as smooth as the surface of an apple. The Lipschitz traces in this experiment (not shown), are again very similar to those in Fig. 6. The original noisy HIM image has *α* = 0.085, while the Lévy (*β* = 0.2) stable image has *α* = 0.211. The *TV* and Curvelet images have significantly higher values of *α*, namely 0.697 and 0.704, respectively. In this example, Lévy (*β* = 0.2) stable denoising provides the microscopist with a more prudent reconstruction, to be considered alongside the *TV* and Curvelet images.

## 12. Short Time Smoothing Using Other Evolution Equations

A significant advantage of the fractional diffusion method is the *slow motion* option which enables monitoring of the denoising process, and the possibility of backtracking to a more optimal image. Equally important is the stopping criterion in the minimum principle in Eq. (23), which provides a user selected parameter *λ* to control the reduction in ║∇*_d_ f*║_2_. When this method is applied to noisy HIM imagery, it is found that a considerable portion of the noise is removed, without significantly altering the original information content of the image. In Figs. 4, 5, 7, 8, 9, and 10, jagged edges in the surface texture, and several other aspects in the original surface morphology, are preserved in the Lévy (*β* = 0.2) stable image. This implies fidelity to high frequency information. In these figures, the value of ║∇*_d_ f*║*_p_*, *p* = 1,2, in the Lévy images was typically reduced by a factor of 3, corresponding to choosing *λ* ≈ 0.33 in Eq. (23).

Such fidelity to the original surface morphology was not feasible with the split Bregman *TV* method, or with the Curvelet Thresholding method. The *TV* method succeeds in its aim at minimizing ║∇*_d_ f*║_1_ in the denoised image. In Figs. 4 and 9, ║∇*_d_ f*║_1_ is reduced by factors of 7 and 13, respectively, after split Bregman *TV* denoising. Unexpectedly, Curvelet denoising produces even more severe reductions in ║∇*_d_ f*║_1_. Clearly, significant fine structure information has been removed, along with the noise, in the *TV* and Curvelet images. This is confirmed by the sizeable increases in image Lipschitz exponents.

Evidently, the stopping criterion in Eq. (23) can be applied to other diffusive equations, and a large variety of methods for image denoising by short time smoothing can be constructed. The heat conduction equation, *w_t_* = ∆*w*, is an obvious candidate to be considered, and it corresponds to choosing *β* = 1 in Eq. (4). Another interesting candidate is the nonlinear Marquina-Osher pde, used in Sec. 10 in lieu of the split Bregman method to implement *TV* denoising. This is

(27) $$\left\{ \begin{array}{l} w_{t}=-\Lambda \left|\nabla w\right|(w-f)+\left|\nabla w\right|\nabla .\left(\nabla w/\{\sqrt{{\left|\nabla w\right|}^{2}+\sigma }\}\right),\\ w(x,y,0)=f(x,y), \end{array}\right.$$

where the given blurred image *f*(*x*, *y*) is used as the initial value. In addition, *w*(*x*, *y*, *t*) satisfies homogeneous Neumann conditions at the boundary of the unit square Ω. In Eq. (27), σ > 0 is a small constant designed to prevent division by zero, and Λ > 0 is a tunable regularization parameter. In [19], an efficient explicit finite difference scheme for Eq. (27) is proposed. This scheme has improved stabilty and edge-enhancing properties, and converges rapidly to the desired steady state solution.

We stress that *TV* denoising requires obtaining the *long time steady-state* solution in Eq. (27), and the split Bregman iteration used in the previous section is merely an alternative method of finding that steady-state. When the Marquina-Osher pde is used for smoothing up to some short time *t*^†^ > 0, the output solution is *unrelated* to either the minimum principle in Eq. (20), or *TV* denoising. However, a short time Marquina-Osher solution may not suffer the severe reduction in ║∇*_d_ f*║_1_ that acccompanies the steady-state solution.

Fractional diffusion denoising exploits the fact that in the evolution equation *w_t_* = −(−Δ)*^β^w*, *t* > 0, *w*(*x*, *y*, 0) = *f*(*x*, *y*), the solution *w*(*x*, *y*, *t*) satisfies the sharp inequality ║∇*w*(.,*t*)║_2_ = *O*(*t*^−1/2^*^β^*), *t* ↓ 0. Thus, if *β* is chosen small, and *f*(*x*, *y*) is not smooth, ║∇*w*(.,*t*)║_2_ blows up much more rapidly as *t* ↓ 0, than would be the case with Gaussian smoothing (*β* = 1). This suggests that for such small *β*, the solution *w_β_*(*x*, *y*, *t*) retains a great deal of the fine structure in the initial data *f*(*x*, *y*), at small values of *t* > 0. Whether and how the choice of *β* actually affects the denoised image is of great interest. These considerations lead to the following experiment.

## 13. Comparing Short Time Smoothing Pdes on a HIM Image

A useful experiment can be devised to test whether equally good, or even better, results might be obtained by applying the stopping criterion in Eq. (23) to other evolution equations. We revisit the noisy original HIM image in Fig. 4, and the corresponding Lévy (*β* = 0.2) denoised image. From Table 1, we see that the fractional diffusion evolution in Eq. (4) with *β* = 0.2, was stopped when ║∇*_d_ f*║_1_ reached the value 8500, corresponding to *t*^†^ = 0.1. Therefore, a meaningful comparison between the Lévy (*β* = 0.2) denoised HIM image in Fig. 4, and corresponding images produced using any other evolution equation, should be based on selecting the output denoised image to be the image obtained when ║∇*_d_ f*║_1_ first reaches the value 8500.

Applying the Marquina-Osher pde in Eq. (27) to the same HIM image, using the finite difference scheme in [19] with Δ*t* = 0.01(Δ*x*)^2^, Λ = 100, and *σ* = 0.0001, we find ║∇*_d_ f*║_1_ = 8500 at *t*^†^ = 6.68E-7. Likewise, applying the heat conduction pde in Eq. (4) with *β* = 1, and using Eq. (14), we find ║∇*_d_ f*║_1_ = 8500 at *t*^†^ = 7.51E-6.

**Fig. 11:**
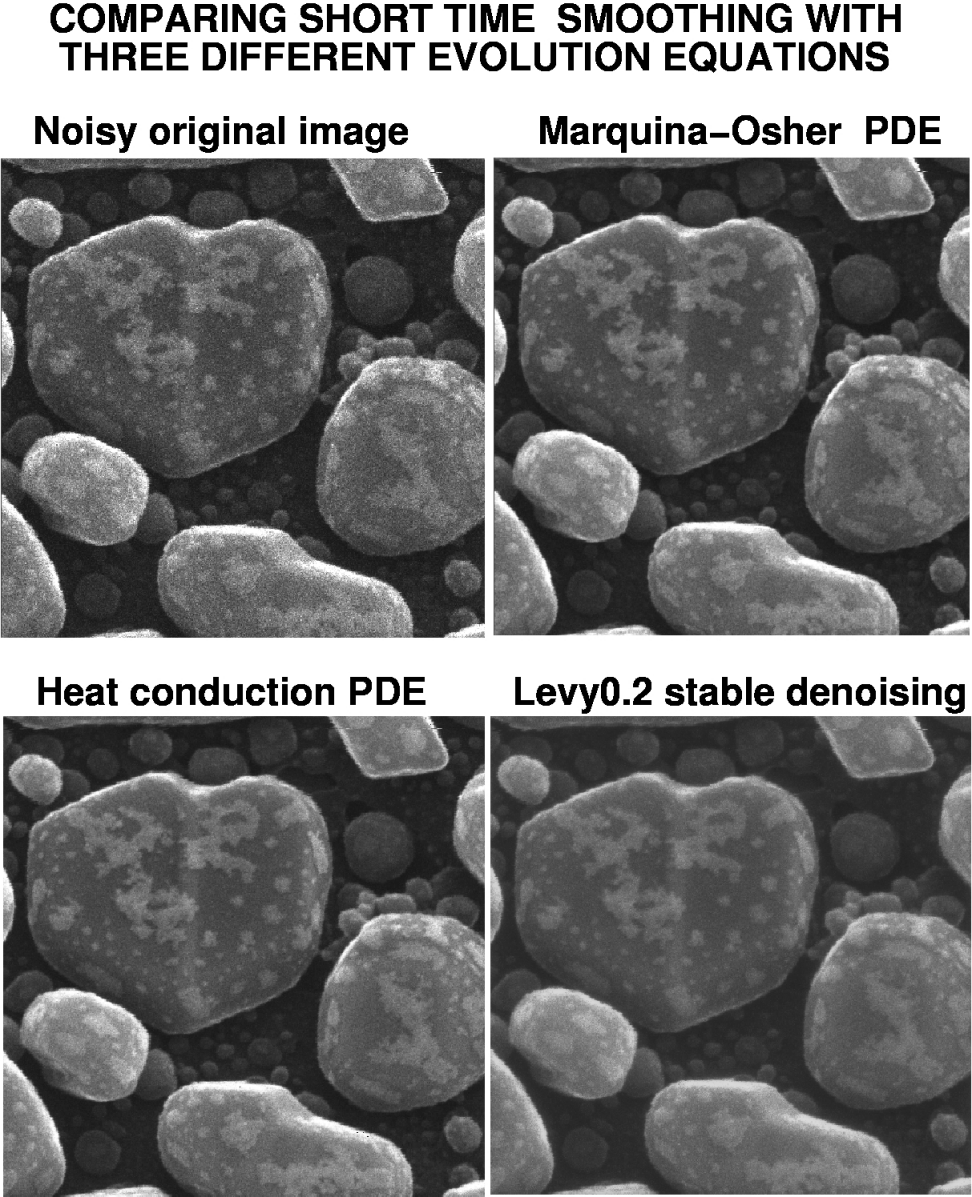
Denoising of HIM Au-decorated gold on carbon sample in Fig. 4, using short time smoothing with three different evolution equations, and stopping the evolution when ║∇*_d_ f*║_1_ first reaches the value 8500. Experienced microscopists find the Lévy (*β* = 0.2) image to be best, followed by the Marquina-Osher and heat conduction pde images. Lipschitz exponents in Fig. 12 concur with this ordering.

The results of this experiment are shown in Fig. 11. The original noisy image and the three candidate denoised images were subjected to individual evaluation by several NIST specialists in high precision microscopy. The particular denoising method used for each image was not disclosed. A summary of their assessment was that while the three processed images showed improvement over the original, the heat conduction pde image was of somewhat lesser quality than the other two. A small minority selected the Marquina-Osher image as the best image. However, the consensus was that the Lévy (*β* = 0.2) denoised image was the best overall.

A more complete picture emerges if we analyze the corresponding Lipschitz traces, after obtaining one additional denoised image, namely the image resulting from using *β* = 0.15 in Eq. (4), and Eq. (14). In this case, we have ║∇*_d_ f*║_1_= 8500 at *t*^†^ = 0.182. The resulting image is a small improvement on the Lévy (*β* = 0.2) image, and is not shown. However, this additional short time smoothing experiment is helpful in establishing a pattern of behavior. The Lévy (*β* = 0.15) image has a smaller Lipschitz exponent, *α* = 0.418, than does the Lévy (*β* = 0.2) image with *α* = 0.451.

**Fig. 12:**
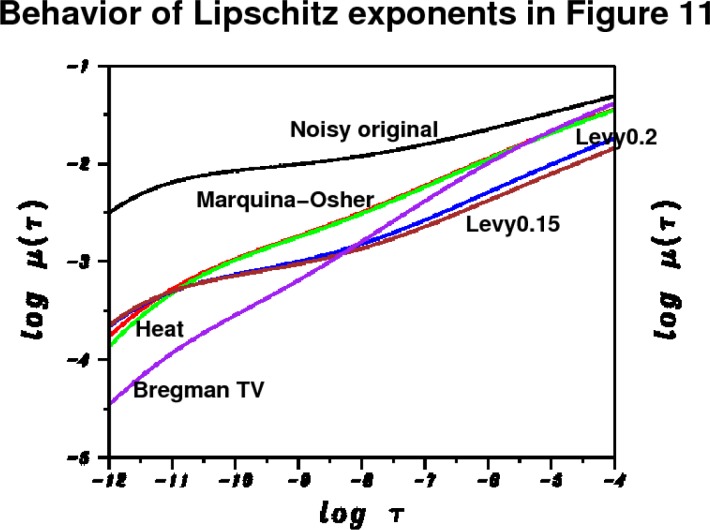
Behavior of *L*^1^ image Lipschitz exponents in short time smoothing experiment on noisy HIM image in Fig. 4, as discussed in Sec. 13 and Fig. 11. Majorizing Σ lines not drawn to avoid clutter. Black curve is original noisy trace with Lipschitz exponent *α* = 0.241. Purple curve corresponds to previously obtained split Bregman TV denoising with Lipschitz exponent *α* = 0.751. Remaining four curves result from short time smoothing using four different evolution equations, and stopping the evolution when ║∇*_d_ f*║_1_ reaches the value 8500. Marquina-Osher pde trace (red) has *α* = 0.520, and almost coincides with heat conduction pde (*β* = 1) trace (green) with *α* = 0.524. Lévy (*β* = 0.2) trace (blue) has *α* = 0.451, and Lévy (*β* = 0.15) trace (brown) has *α* = 0.418. Above behavior pattern is replicated when similar experiments are performed on noisy HIM images in Figs. 7 and 9. These experiments indicate that decreasing the Lévy exponent *β* in Eq. (4) also decreases the resulting Lipschitz exponent *α*, if smoothing is stopped at the same value of ║∇*_d_ f*║_1_.

The Lipschitz traces for Fig. 11, together with the Bregman *TV* trace (purple) from Fig. 6, and the Lévy (*β* = 0.15) trace (brown), are shown in Fig. 12. The Marquina-Osher (red) and heat conduction (green) images have almost identical Lipschitz traces, and these traces are noticeably different from the Bregman *TV* trace. Clearly, use of the stopping criterion Eq. (23) in the Marquina-Osher pde, results in significant improvement over the use of the *TV* minimum principle, Eq. (20). As seen in Table 4, not only is the Lipschitz exponent *α* = 0.52 substantially smaller than was the case in Bregman *TV* with *α* = 0.75, but the L^1^ norm has been preserved as well.

Another significant observation in Fig. 12 and Table 4, is that the Lipschitz exponent is a mathematical index that captures image small-scale features in a more subtle way than does ║∇*_d_ f*║_1_. The red, green, blue, and brown traces in Fig. 12 are associated with four different versions of the same HIM image, in each of which ║∇*_d_ f*║_1_ has the identical value 8500. Apparently, for a fixed exit value of ║∇*_d_ f*║_1_, the resulting Lipschitz exponent *α* depends primarily on the (fractional) order of the spatial differential operator in the diffusive evolution equation. Thus, both the linear isotropic heat conduction pde, and the nonlinear anisotropic Marquina-Osher pde, result in almost identical Lipschitz traces. The green, blue, and brown traces indicate that decreasing the order of the spatial operator by decreasing *β* in Eq. (4), also decreases the resulting Lipschitz exponent *α*. Significantly, this behavior pattern is *replicated* when the above experiment is repeated on the noisy HIM images in Figs. 7 and 9. Remarkably, the short time Marquina-Osher and heat conduction pdes again result in almost identical Lipschitz traces, with larger Lipschitz exponents than in the Lévy (*β* = 0.2) and Lévy (*β* = 0.15) traces.

It is recognized that end-user perception of quality and value in the processed image is often the final arbiter of usefulness in a proposed image processing methodology. However, the above experiments indicate that as suggested by the inequality in Eq. (13), the use of fractional diffusion equations can be advantageous in image denoising.

## Figures and Tables

**Table 1:** Behavior of ║*f_d_*║_1_, ║*f_d_*║_2_, ║∇*_d_ f*║_1_, and Lipschitz *α*, in Fig. 4 denoising. Note severe ║∇*_d_ f*║_1_ reduction in Curvelet and *TV* denoising.

Image *f* (*x*, *y*)	║*f_d_*║_1_	║*f_d_*║_2_	║∇*_d_ f*║_1_	Lip *α*
Noisy original (600 nm)	88	99	25000	0.241
Lévy stable (*β* = 0.2, *t*^†^ = 0.1)	88	94	8500	0.451
Split Bregman TV (*ω* = 0.025)	74	89	3400	0.751
Curvelet thresholding (*σ_n_* = 30)	81	91	2700	0.810

**Table 2:** Behavior of ║*f_d_*║_1_, ║*f_d_*║_2_, ║∇*_d_ f*║_1_, and Lipschitz *α*, in Fig. 7 denoising. Note severe ║∇*_d_ f*║_1_ reduction in Curvelet and *TV* denoising.

Image *f* (*x*, *y*)	║*f_d_*║_1_	║*f_d_*║_2_	║∇*_d_ f*║_1_	Lip *α*
Noisy original (90 *μ*m)	54	65	14000	0.299
Lévy stable (*β* = 0.2, *t*^†^ = 0.1)	54	61	4900	0.497
Split Bregman TV (*ω* = 0.025)	37	57	3000	0.638
Curvelet thresholding (*σ_n_* = 30)	46	56	2600	0.668

**Table 3:** Behavior of ║*f_d_*║_1_, ║*f_d_*║_2_, ║∇*_d_ f*║_1_, and Lipschitz *α*, in Fig. 9 denoising. Note severe ║∇*_d_ f*║_1_ reduction in Curvelet and *TV* denoising.

Image *f* (*x*, *y*)	║*f_d_*║_1_	║*f_d_*║_2_	║∇*_d_ f*║_1_	Lip *α*
Noisy original (300 nm)	74	84	47000	0.085
Lévy stable (*β* = 0.2, *t*^†^ = 0.1)	74	78	15000	0.211
Split Bregman TV (*ω* = 0.025)	73	82	3500	0.697
Curvelet thresholding (*σ_n_* = 30)	64	70	3000	0.704

**Table 4:** Behavior of ║*f_d_*║_1_, ║*f_d_*║_2_, ║∇*_d_ f*║_1_, and Lipschitz *α*, in Fig. 11 denoising.

Image *f* (*x*, *y*)	║*f_d_*║_1_	║*f_d_*║_2_	║∇*_d_ f*║_1_	Lip *α*
Noisy original (600 nm)	88	99	25000	0.241
Lévy stable (*β* = 0.15, *t*^†^ = 0.182)	88	93	8500	0.418
Lévy stable (*β* = 0.2, *t*^†^ = 0.1)	88	94	8500	0.451
Marquina-Osher pde (*t*^†^ = 6.68E-7)	87	97	8500	0.520
Heat conduction eqn. (*t*^†^ = 7.51E-6)	88	98	8500	0.524
Split Bregman TV (*ω* = 0.025)	74	89	3400	0.751
